# U three protein 14a (UTP14A) promotes tumour proliferation and metastasis via the PERK/eIF2a/GRP78 signalling pathway in oesophageal squamous cell carcinoma

**DOI:** 10.7150/jca.44649

**Published:** 2021-01-01

**Authors:** Kun-Kun Li, Cheng-Yi Mao, Qiang Ma, Tao Bao, Ying-Jian Wang, Wei Guo, Xiao-Long Zhao

**Affiliations:** 1 Department of Thoracic Surgery, Daping Hospital, Army Medical University, PR China.; 2 Department of Pathology, Daping Hospital, Army Medical University, PR China.

**Keywords:** UTP14A, PERK, ESCC, metastasis

## Abstract

Metastasis and malignant proliferation are major obstacles to the treatment of oesophageal squamous cell carcinoma (ESCC), and UTP14A is associated with poor prognosis in ESCC. However, its mechanisms have not been fully elucidated. The TCGA and GEO databases were used to identify candidate target genes and possible downstream targets. Then, the effects were determined *in vitro* and *in vivo* through knockdown and overexpression techniques, and the mechanism was explored. UTP14A was significantly higher in the tumour tissue of ESCC patients than in normal tissue. Knockdown of UTP14A significantly suppressed the migration and proliferation of ESCC cells. The PERK/eIF2a signalling pathway was positively regulated by UTP14A, and its tumour-promoting effect was further activated by overexpression of UTP14A. In conclusion, UTP14A might promote the proliferation and metastasis of ESCC cells by inducing PERK/eIF2a signalling pathway expression.

## Introduction

Oesophageal cancer is a common malignant tumour in China, but most patients are already in the advanced stage at the time of diagnosis and have passed the optimal time for surgery. Therefore, comprehensive treatment with chemoradiation has become an indispensable treatment method. Although treatment has improved in recent years, the prognosis of patients with oesophageal cancer is still not optimistic, and the 5-year survival rate is only 15.0% to 20.9% 1. Early invasion and metastasis of tumour cells are the main reasons for poor prognosis in patients with ESCC (oesophageal squamous cell carcinoma); therefore, it is necessary to explore the possible mechanisms of oesophageal cancer invasion and metastasis 2, 3. In addition, effective and sensitive biomarkers and new therapeutic targets have not yet been identified.

Previous studies have demonstrated that UTP14A plays a key role in the synthesis of ribosomes and 18S rRNA 4, 5. Hu et al. reported that UTP14A is overexpressed in various kinds of tumour cells 6. The expression of UTP14A is associated with poor prognosis in human hepatocellular carcinoma and colorectal carcinoma 7, 8. Our previous study also demonstrated that higher expression of UTP14A is a predictor of poor prognosis in patients with ESCC 9. However, the mechanism of how UTP14A affects the prognosis of patients with oesophageal cancer remains unclear.

In this study, we investigated the mechanism by which UTP14A promotes tumour progression in ESCC. A new gene was found to be involved in the regulation of ESCC metastasis, and its possible mechanism was explored.

## Materials and methods

### Human specimens and cell lines

Human specimens were obtained from ESCC patients by biopsy or surgery at Daping Hospital. This study was approved by the Ethics Committee of Daping Hospital, Army Medical University. HEEC, TE-1, KYSE-70, Eca109 and KYSE-150 cells were obtained from American Type Culture Collection (ATCC, Manassas, VA, USA).

### Plasmids and transfection

The UTP14A ORF was amplified by PCR from human cDNA and inserted into the Bam HI and Hind III sites of the pcDNA3.1-flag vector (GeneChem Shanghai, China) for expression in ESCC cells. For small interfering RNA (shRNA) transfections, Eca109 cells were infected with UTP14A-shRNA lentivirus and NC lentivirus (GeneChem Shanghai, China). The shRNA primers are shown in **Table 1.**

### Cell viability assay

The cell viability assay was performed according to the manufacturer's protocol. Cells were plated in a 96-well plate. After 12 h of cell seeding, cells were treated with the indicated drugs for 48 h, and cell viability was determined using a CCK-8 kit (Dojindo Laboratories, Kumamoto, Japan) 10.

### RNA sequencing array

First, rRNAs in samples from the control and PM2.5 groups were removed. Then, the libraries for next-generation sequencing were prepared using the Truseq RNA sample prep Kit (Illumina, USA). After enrichment and purification, the libraries were processed for sequencing by Shanghai Origingene Bio-pharm Technology Co., Ltd. (Shanghai, China) according to an available protocol. After quality control of the original data, the high-quality sequencing data were compared with the designated reference genome. The expression values were calculated by the StringTie tool, and the tDESeq algorithm was applied to filter the differentially expressed genes.

### Apoptosis analysis

ESCC cells were transfected with the shRNA or plasmids. After 48 h of transfection, cells were harvested and stained with annexin V and 7-aminoactinomycin D (7-AAD) and then subjected to flow cytometric analysis 9.

### Immunohistochemistry (IHC) and immunofluorescence (IF)

IHC and IF were performed as described previously 11. Sections were observed using an upright phase contrast light microscope (Nikon Corporation, Tokyo, Japan). The IHC staining score was reviewed by an expert panel of pathologists. Primary antibodies (anti-PERK, Abcam Cat. no. ab65142; anti-GRP78, Abcam Cat. no. ab21685; anti-Ki-67 Promab Cat. no. 30100), and secondary antibodies were obtained from Abcam.

### Western blot

Western blotting was performed as described previously 11, followed by incubation with the appropriate primary antibodies (anti-UTP14A, Proteintech Cat. no. 11474-1-AP; anti-PERK, Abcam Cat. no. ab65142; anti-ATF4, Abcam Cat. no. ab184909; anti-eIF2a, Promab Cat. no. 30599; anti-p-eIF2a, CST Cat. no. #9721; and anti-GRP78, Abcam Cat. no. ab21685) 11.

### Invasion assay

The invasion assay was performed as described previously 9**.** Cells were transfected with the UTP14A expression plasmid or shRNA targeting UTP14A (GeneChem Co., Shanghai, China) using Lipofectamine 3000 (Invitrogen, Carlsbad, CA, USA) according to the manufacturer's instructions. At 48 h post transfection, the cells were subjected to an invasion assay. After 24 h of cell seeding, the cells that had invaded the lower side of the chamber were fixed with 2.5% glutaraldehyde, stained with 0.1% crystal violet, and counted 9.

### Animal experiments

All xenograft models were generated using Eca109 cells in 6-week-old female BALB/c nude mice. Eca109 cells (1×10^6^/50 μl per mouse) were transfected with non-targeting control shRNA, shUTP14A and a plasmid overexpressing UTP14A. Eca109 cells were suspended in Matrigel and injected subcutaneously (s.c.) into each mouse (right back). The experimental procedures were approved by the Daping Hospital, Army Medical University Institutional Animal Care and Use Committee. Following implantation, tumour volumes were measured every 7 days until the mice were sacrificed by CO_2_ at day 35.

### Statistical analyses

All statistical analyses were performed using Prism 6.0 software (GraphPad). The independent sample *t* test was used for comparing groups to determine significant differences, and *P* values less than 0.05 were considered statistically significant. The data are presented as the mean with the standard deviation (SD) of at least three independent experiments. The Pearson's correlation coefficient was obtained with the R language package corrplot.

## Results

### UTP14A is highly expressed in oesophageal squamous cell carcinoma

**Figure 1:**
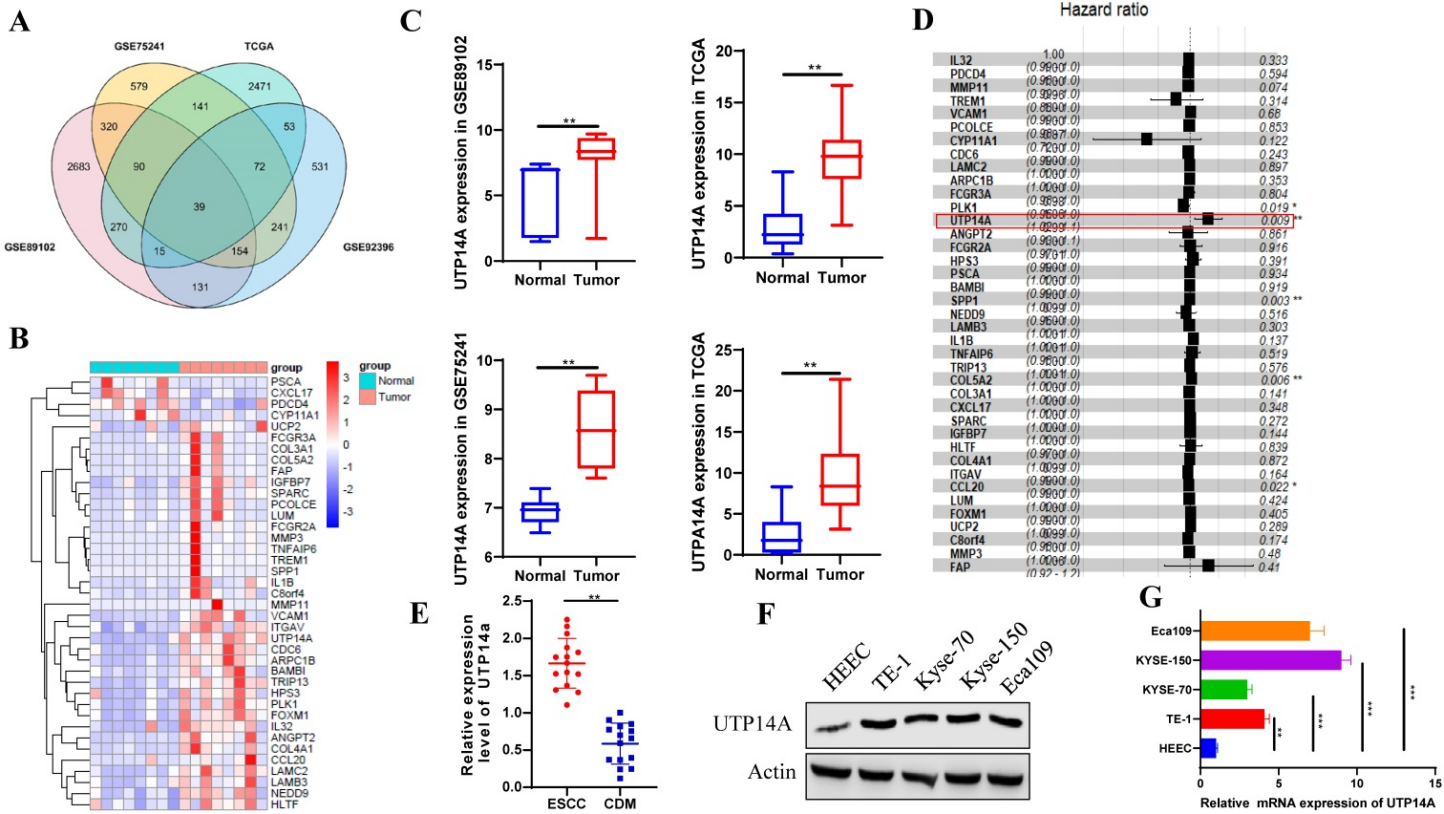
** ESCC patients with lymph node metastasis have higher expression of UTP14A. A.** The GEO and TCGA databases were used to analyse differentially expressed genes in oesophageal cancer and normal oesophageal tissue, and 39 genes were found to be significantly different. **B.** The heat map shows the expression of 39 genes upregulated in ESCC patients compared to normal controls. **C.** UTP14A expression was upregulated in ESCC patients compared to normal controls. **D.** Cox regression analysis showed the risk of ESCC. **E.** The expression of UTP14A in ESCC and CDM detected by RT-PCR. **F.** Relative expression of UTP14A in various common tumour cell lines detected by western blot. **G.** Relative expression of UTP14A in various common tumour cell lines detected by RT-PCR. Values are presented as the means ± standard deviations of three independent experiments unless otherwise indicated. CDM, cancer distant mucosa. * *P*<0.05, ** *P*<0.01, *** *P*<0.001.

We used the GEO and TCGA databases to analyse differentially expressed genes in oesophageal cancer and normal oesophageal tissue. The resulting Venn diagram showed that there were 39 genes that were significantly different in both the GEO and TCGA databases (Fig. 1A). The heat map showed that the expression of the 39 genes was significantly different and UTP14A expression was upregulated in ESCC patients compared to normal patients (Fig. 1B, C). Cox regression analysis showed that UTP14A is a risk factor for ESCC (*P=*0.009, Fig. 1D). Then, we used tissues from 15 ESCC patients at our hospital and found that UTP14A expression was significantly higher in ESCC samples than in CDM (cancer-distant mucosa) samples (Fig. 1E). The data showed that UTP14A may play a key role in ESCC progression. To further study the role and mechanism of UTP14A in tumour metastasis by *in vitro* experiments, we tested the protein and mRNA expression of UTP14A in several commonly used ESCC cell lines, among which KYSE-150 cells and Eca109 cells had relatively higher expression (Fig. 1F, G).

### UTP14A promotes cell proliferation and migration

**Figure 2:**
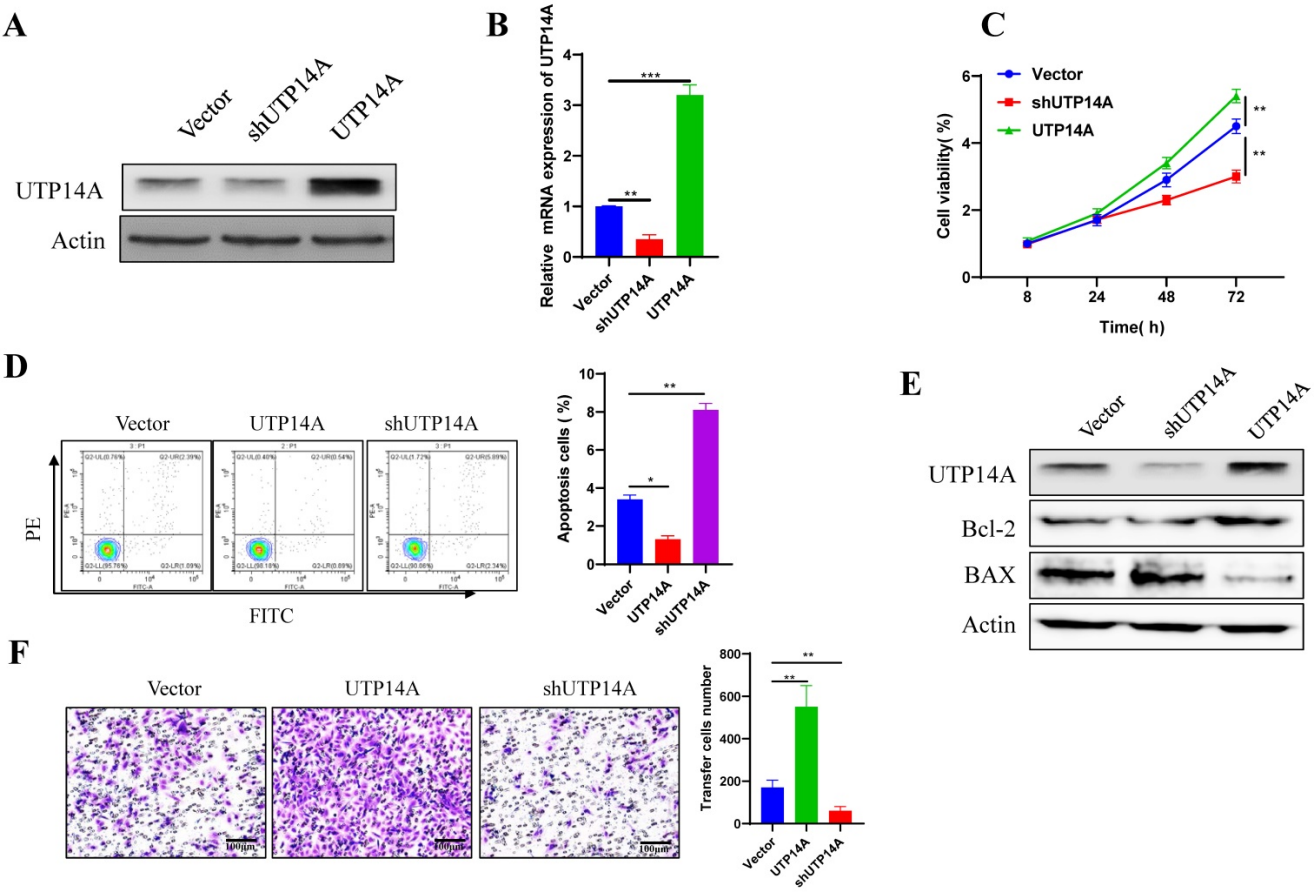
** UTP14A enhanced Eca109 cell proliferation and migration. A.** Eca109 cells with relatively high UTP14A expression levels were used for *in vitro* overexpression and knockdown experiments, and protein levels were detected by western blotting. **B.** Eca109 cells were used for *in vitro* overexpression and knockdown experiments, and expression levels were detected by RT-PCR. **C.** The effect of UTP14A on cell viability was detected by the CCK-8 assay. **D.** Flow cytometry analysis of the effect of UTP14A on apoptosis-related proteins. **E.** Western blot analysis of the effect of UTP14A on apoptosis-related proteins. **F.** Invasion analysis of the effect of UTP14A on migration. * *P*<0.05, ** *P*<0.01, *** *P*<0.001.

To further clarify the mechanism of UTP14A in tumour cell growth and metastasis, Eca109 cells, which had relatively high expression levels of UTP14A, were used for overexpression and knockdown experiments *in vitro* (Fig. 2A, B). Compared with the non-target control (NC) shRNA group, cell viability was found to be significantly inhibited in the UTP14A knockdown group, while overexpression of UTP14A significantly enhanced cell viability (Fig. 2C). Since UTP14A plays a role in regulating Eca109 cells viability, the effect of UTP14A on tumour cell apoptosis was further investigated through flow cytometry. When downregulation of UTP14A significantly increased the apoptotic rate of tumour cells, while overexpression of UTP14A reduced apoptosis (Fig. 2D). Western blot analysis also showed similar results: knockdown of UTP14A significantly increased Bax and suppressed Bcl-2, while UTP14A overexpression showed the opposite result (Fig. 2E). Subsequent scratch experiments showed that overexpression of UTP14A significantly enhanced the migration of Eca109 cells, while UTP14A knockdown inhibited this migration (Fig. 2F).

### Transcriptome sequencing analysis of the downstream pathway of UTP14A

**Figure 3:**
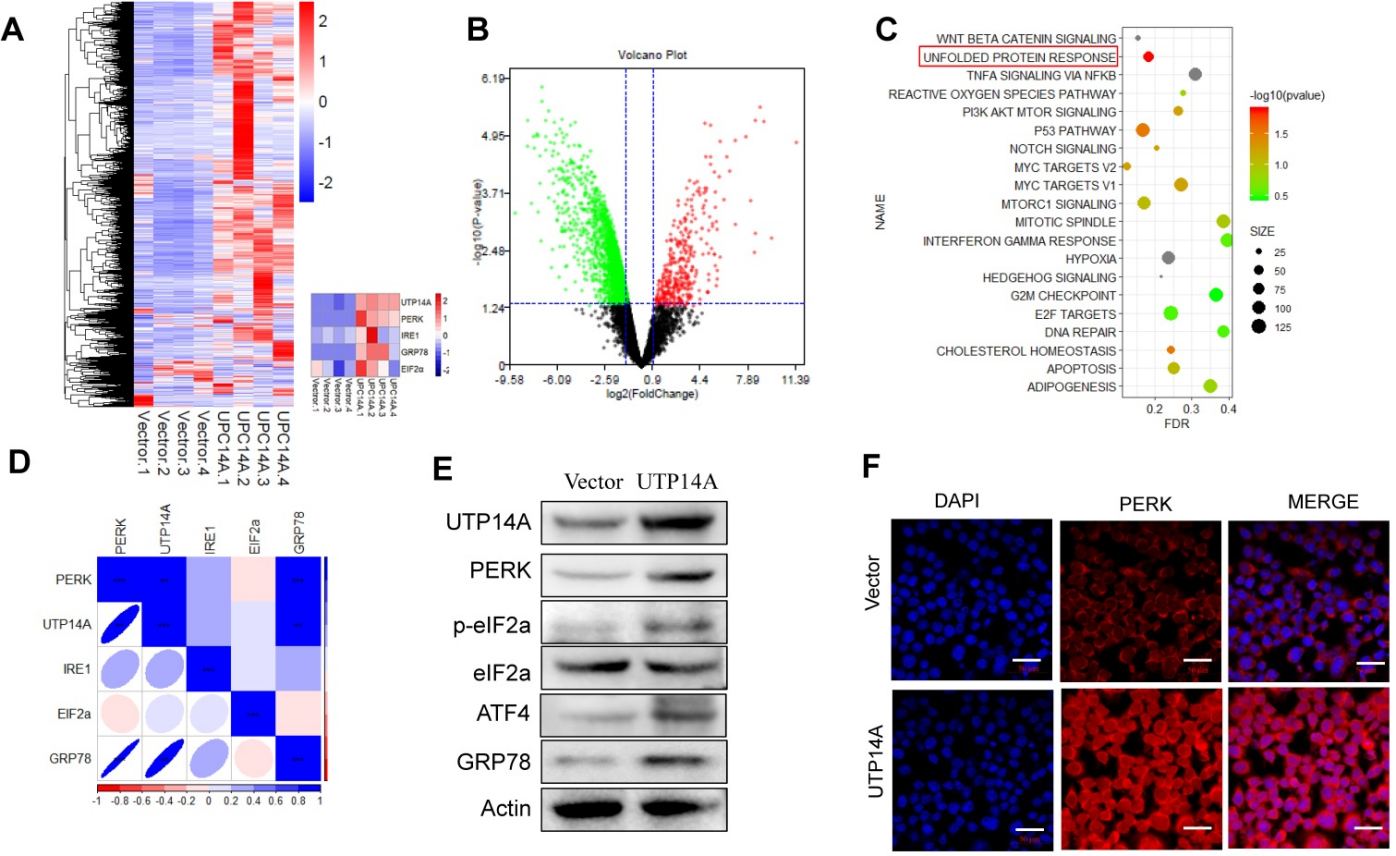
** UTP14A regulates the downstream pathway PERK/eIF2a. A and B.** The heat map and volcano map show differential gene expression. **C.** KEGG analysis of UTP14A high-expression and low-expression pathway differences. **D.** Correlation analysis between UTP14A and unfolded protein response pathway protein expression. **E.** Western blotting was performed to determine the effect of UTP14A on the expression of PERK, eIF2a and GRP78. **F.** Immunofluorescence was used to determine the effect of UTP14A on the expression of PERK. ** *P*<0.01, *** *P*<0.001.

We transfected UTP14A into Eca109 cells for 72 h and performed a transcriptome sequencing analysis of the downstream pathway of UTP14A. From the sequencing analysis, the heat map and volcano map showed the differential gene expression and the UTP14A significant difference (Fig. 3A, B). Then, we analysed the UTP14A high-expression and low-expression pathway differences by KEGG analysis, and the data showed that the unfolded protein response pathway was significantly different (Fig. 3C).

The correlation analysis showed a correlation between UTP14A levels and the expression of unfolded protein response pathway proteins, such as PERK, eIF2a and GRP78 (Fig. 3D). Western blot analysis also showed similar results, whereby overexpression of UTP14A upregulated the expression of PERK, eIF2a and GRP78 (Fig. 3E). We also confirmed this result using immunofluorescence, which showed that UTP14A could upregulate the expression of PERK (Fig. 3F).

### UTP14A promotes Eca109 cell growth and migration by the PERK signalling pathway

**Figure 4:**
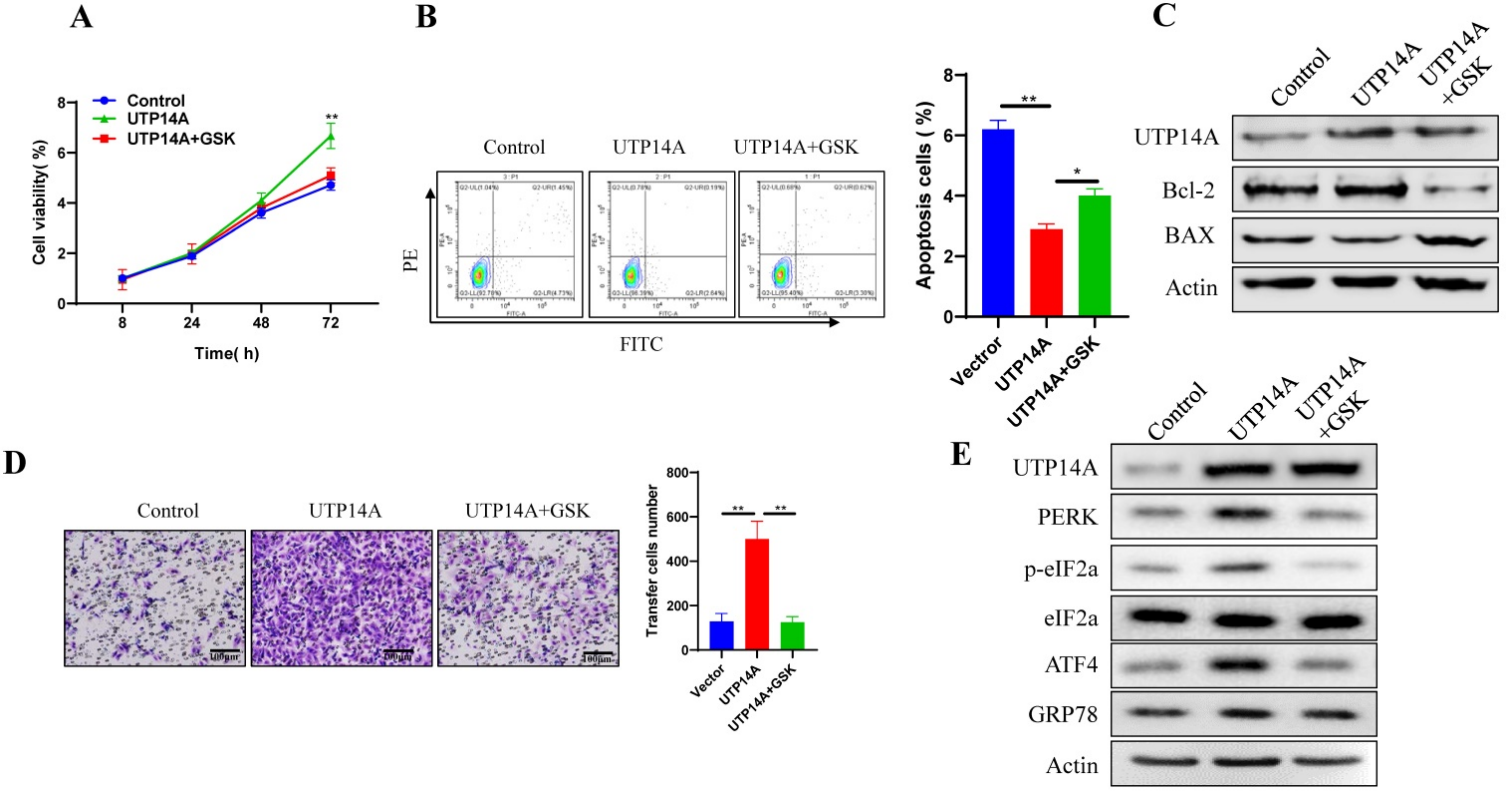
** UTP14A inhibits ESCC via the PERK signalling pathway *in vitro*. A.** Inhibition of PERK significantly prevented UTP14A from promoting cell growth, as detected by the CCK-8 assay. **B.** Inhibition of PERK significantly prevented UTP14A from suppressing cell apoptosis, as detected by flow cytometry. **C.** Inhibition of PERK significantly blocked the effect of UTP14A on the expression of apoptosis-related proteins (BCL2 and BAX), as detected by western blot. **D.** Inhibition of PERK significantly prevented UTP14A from promoting cell migration, as detected by invasion assay. **E.** Inhibition of PERK significantly blocked the effect of UTP14A on the expression of the PERK/eIF2a signalling pathway, as detected by western blot. * *P*<0.05, ** *P*<0.01.

The above results showed that UTP14A could promote ESCC cell growth and migration, which could be inhibited by suppressing PERK expression. Next, we used GSK2606414 to inhibit PERK expression and investigated whether UTP14A regulates tumour growth through PERK. First, we selected a suitable concentration (2 μM) of GSK2606414 (Fig. S1B). To further verify whether the effect of UTP14A on ESCC cells was achieved by regulating the expression of PERK, we observed the effects of PERK knockdown on tumour cells and whether this effect was regulated by UTP14A. As shown in Fig. 4A, PERK inhibition significantly prevented UTP14A from promoting cell growth. Furthermore, downregulating PERK expression by GSK2606414 significantly promoted apoptosis, which was partially blocked by simultaneous overexpression of UTP14A (Fig. 4B). Further investigation found that GSK2606414 may promote apoptosis of ESCC cells through downregulation of Bcl-2 expression and upregulation of Bax (Fig. 4C). Furthermore, the migration assay (Fig. 4D) also showed similar results. UTP14A overexpression upregulated the unfolded protein response pathway, but GSK2606414 blocked the expression of the unfolded protein response pathway induced by UTP14A (Fig. 4E). The promoting effect of UTP14A on unfolded protein response pathway expression also needs to be confirmed *in vivo*.

### Knockdown of UTP14A inhibits ESCC cell growth *in vivo*

**Figure 5:**
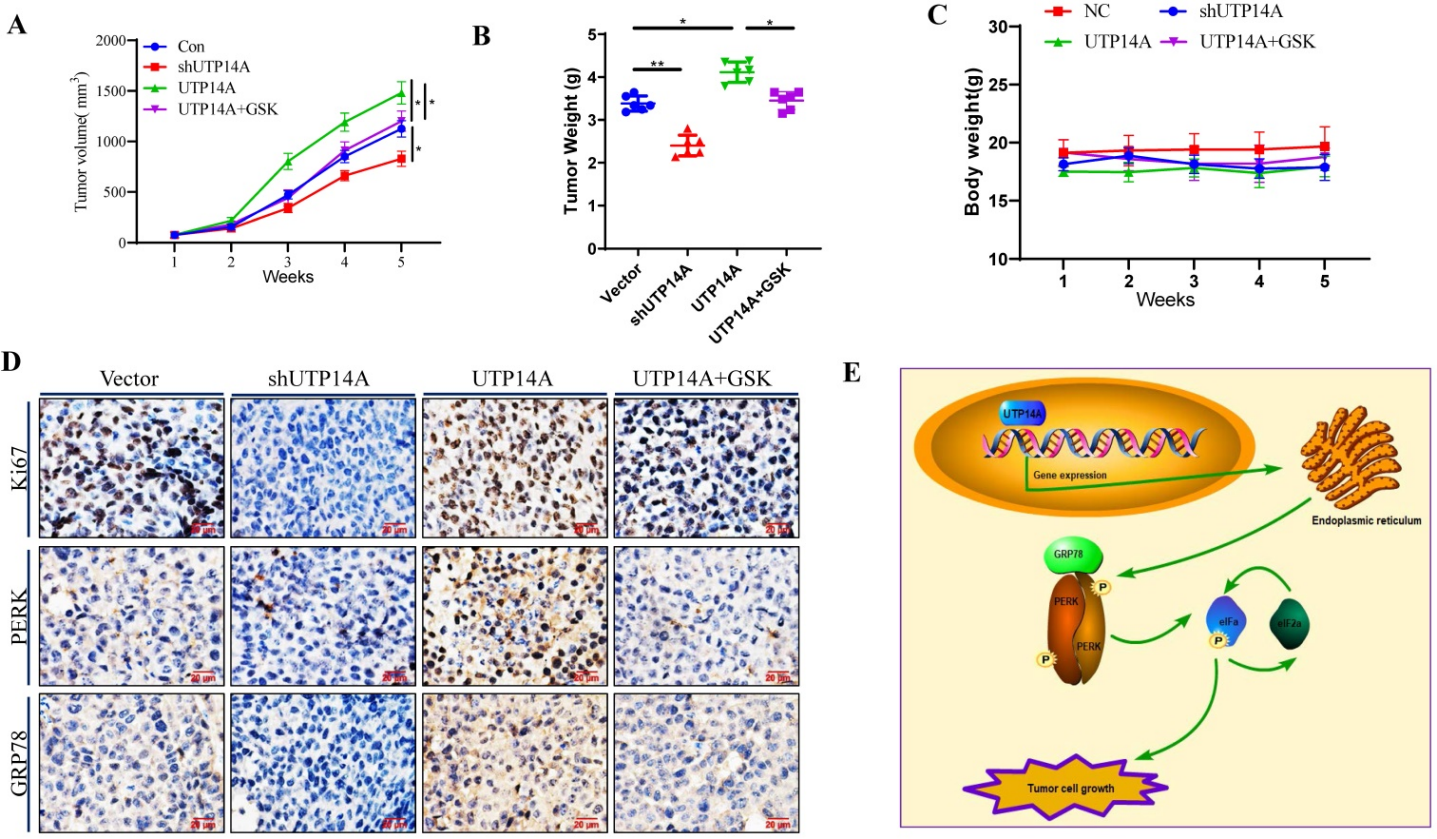
** UTP14A inhibits ESCC growth via the PERK signalling pathway *in vivo*. A.** Tumour volume in xenografts. Eca109 cells were transfected with the UTP14A and shUTP14A plasmids or treated with the PERK inhibitor GSK2606414. **B.** Xenograft weight.** C.** The mouse body weight. **D**. Immunohistochemistry assays of Ki-67, PERK, and GRP78 expression *in vivo* (100×). **E**. A schematic model showing that UTP14A is essential to promote tumour growth via the PERK pathway *in vivo* and *in vitro*. * *P*<0.05, ** *P*<0.01.

In this study, UTP14A-deficient Eca109 cells treated with shRNA and UTP14A-overexpressing Eca109 cells were administered subcutaneously, and their tumorigenesis was observed at the same time. As depicted in Fig. 5A, the growth of UTP14A-deficient cancer cells was significantly reduced, while the growth of UTP14A-overexpressing tumours was significantly enhanced compared with that of the control. Furthermore, GSK2606414 could block the tumour growth induced by UTP14A, similar to the *in vitro* experiments. The weight of the tumours was also reduced in the UTP14A knockdown group (Fig. 5B). No significant difference in mouse weight was observed (Fig. 5C). The expression of Ki67 in various groups of cancer cells showed similar results, and GSK2606414 blocked the PERK/GRP78 expression induced by UTP14A *in vivo* (Fig. 5D). Taken together, these data suggest that UTP14A is essential to promote tumour growth via the PERK pathway *in vivo* and *in vitro* (Fig. 5E).

## Discussion

Metastasis is the main cause of the low curative rate of oncologic tumours 12. ESCC is a complex illness, and many elements are involved in its occurrence. Several investigators have proposed that UTP14A is overexpressed in various kinds of tumours 8, 9, 13. UTP14A has been found to be associated with poor prognosis in ESCC patients 9. In this study, the UTP14A regulatory mechanism was described for the first time in ESCC patients. ESCC cells with UTP14A overexpression or knockdown were used to investigate its impact on ESCC cell proliferation, apoptosis and migration. Our data showed that UTP14A might promote ESCC cell growth and migration by inhibiting the PERK/eIF2a signalling pathway in ESCC cells. The UPR PERK/eIF2a signalling pathway has been reported in tumour apoptosis, migration and tumour angiogenesis 14, 15. Protein imbalance appears to be a hallmark of cancer development and metastasis. Increasing evidence suggests that the endoplasmic reticulum (ER) is an organelle that is involved in protein folding and protein quality control and plays a vital role during this period 14, 16, 17. The accumulation of unfolded or misfolded proteins in the ER caused by perturbations leads to a cytoprotective response called the unfolded protein response (UPR). The three key signalling pathways related to UPR (IRE1α, PERK and ATF6) participate in the response to ER stress by enhancing ER function. Endoplasmic reticulum function increases during cancer cell proliferation, protein synthesis increases, and ER chaperone protein promotes cancer cell survival and invasion 14. Our sequencing results revealed a correlation between UTP14A and the UPR pathway. Therefore, we further used the PERK inhibitor GSK2606414 to verify the regulatory effect of UTP14A on the PERK/eIF2a pathway. These data suggest that UTP14A is essential to promote tumour growth via the PERK pathway *in vivo* and *in vitro*.

In summary, this study found that UTP14A acts as a promoter in ESCC metastasis and proliferation, which may be achieved through the PERK/eIF2a signalling pathway and its induced expression.

## Supplementary Material

Supplementary figures and tables.

## Figures and Tables

**Table 1:** The primer sequences information

Pyrosequencing primers	Sequence (5'-3')
UTP14a Forward	AATAAAACCGCACAAGTCC
UTP14a Reverse	ACAGGGGTCAGTAAAGGGT
GAPDH Forward	TGACTTCAACAGCGACACCCA
GAPDH Reverse	CACCCTGTTGCTGTAGCCAAA
UTP14A shRNA	GAGCAGCTGCGGAAGGTTAAT
