# Supplementary material for: U three protein 14a (UTP14A) promotes tumour proliferation and metastasis via the PERK/eIF2a/GRP78 signalling pathway in oesophageal squamous cell carcinoma
Source: J Cancer. 2021 Jan 1;12(1):134–40. doi: 10.7150/jca.44649 (PMC7738832; doi:10.7150/jca.44649)
Supplement: Supplementary file 1 — Supplementary figures and tables. [file jcav12p0134s1.pdf]

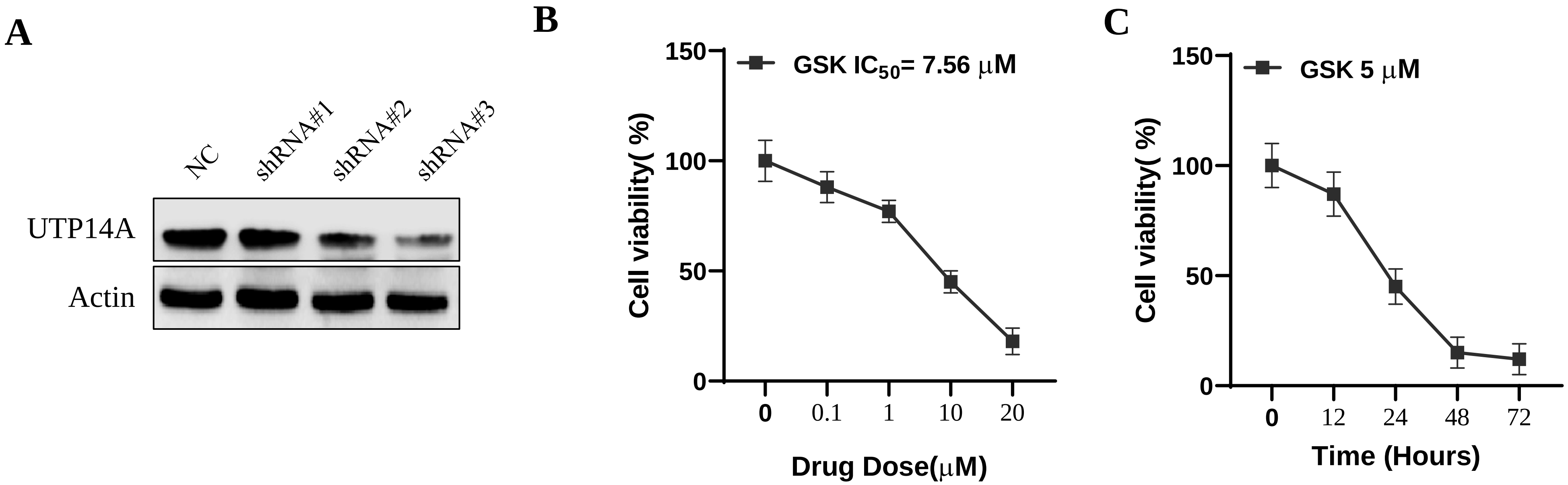
A
NC
shRNA#1
shRNA#2
shRNA#3
UTP14A
Actin
B
GSK IC50= 7.56 μM
Cell viability(%)
150
100
50
0
0
0.1
1
10
20
Drug Dose(μM)
C
GSK 5 μM
Cell viability(%)
150
100
50
0
0
12
24
48
72
Time (Hours)

FigureS1

Supplementary Figure 1. A. The shRNA knocks down UTP14A detected by western blotting. **B.** Inhibition of esophageal cancer cells (Eca109) with different concentrations of GSK, detected by CCK8. **C.** 5μM GSK inhibits esophageal cancer cells (Eca109) at different times, detected by CCK8.
